# Your Mileage May Vary: Individuals with Primary Progressive Aphasia Differ Widely in Their Utilization of Congruent Prosodic and Visual Information During Sentence Comprehension

**DOI:** 10.3390/brainsci16020149

**Published:** 2026-01-29

**Authors:** Mathew Chaves, Marco A. Lambert, Lindsey Kelly, Isidora Diaz-Carr, Voss Neal, Argye E. Hillis, Melissa D. Stockbridge

**Affiliations:** 1 Department of Neuroscience, Krieger School of Arts and Sciences, Johns Hopkins University, Baltimore, MD 21205, USA; 2 Department of Neurology, Cerebrovascular Division, School of Medicine, Johns Hopkins University, Baltimore, MD 21287, USA; 3 Department of Physical Medicine & Rehabilitation, School of Medicine, Johns Hopkins University, Baltimore, MD 21287, USA; 4 Department of Cognitive Science, Krieger School of Arts and Sciences, Johns Hopkins University, Baltimore, MD 21218, USA

**Keywords:** aphasia, dementia, progressive aphasia, comprehension, prosody, expression

## Abstract

**Background/Objectives:** Primary progressive aphasia (PPA) is a clinical syndrome associated with gradual language impairment caused by neurodegenerative disease. While people with post-stroke aphasia often depend on visual and prosodic cues to facilitate language, we hypothesized that people with PPA may have difficulty using such cues due to degeneration in the right hemisphere (albeit less than in the left hemisphere) in PPA. **Methods:** Eighty-eight outpatients diagnosed with PPA received the Hopkins Auditory Comprehension with Context Assessment (HACCA), a recently developed instrument that systematically titrates both acoustic (prosody) and visual (speaker image) cues in a four-item forced-choice sentence picture matching paradigm assessing comprehension. Patients were grouped based on the effects of cues on accuracy and were examined both by the PPA variant and individually. **Results:** There was a significant difference between performance classifications across the three cueing conditions as a function of PPA variant (*p* = 0.014). When individuals with distinct complementary profiles of performance across conditions were examined separately, a small number with logopenic PPA uniquely benefitted from the inclusion of video, while certain patients performed more poorly given any additional cues. HACCA performance across cueing conditions had a strong positive association with other concurrent measures of communication and cognition. **Conclusions:** Individual patterns of response to prosodic and visual cues provide important insights valuable in refining therapeutic approaches that target the retention of function and support a more robust understanding of the individual variability among patients with this uncommon neurodegenerative syndrome.

## 1. Introduction

Primary Progressive Aphasia (PPA) is an uncommon communication disorder characterized by a gradual loss of language. It can be caused either by frontotemporal lobar degeneration (FTLD) or by Alzheimer’s disease (AD). PPA has three variants: logopenic (lvPPA, often associated with AD), nonfluent/agrammatic (nfavPPA), and semantic (svPPA), with the latter two typically associated with FTLD. The characterization of each of the main variants was outlined in a consensus statement published in 2011 [1]. Distinct patterns of atrophy are associated with each of the three primary PPA variants [2,3]. Individuals with lvPPA typically exhibit atrophy in the left temporoparietal region [4]. In contrast, patients with nfavPPA tend to show atrophy in the left inferior frontal gyrus and insula, and those with svPPA have the greatest atrophy in the left anterior temporal lobe. However, these patterns of atrophy characterize the dominant localization of atrophy, not exclusive or exhaustive areas, and all three variants show bilateral atrophy (the left greater than the right) [3].

Effective communication involves far more than retrieving words and constructing grammatical sentences. In addition to the message encoded by the words, spoken communication involves prosody, the vocal intonation patterns of rhythm, pitch, duration, and stress, which shape discourse [5]. Prosody has different functions, which can be used simultaneously to build a message. These functions may include conveying part of speech (e.g., the verb “obJECT” versus the noun “OBject”), indicating when something is a question versus a statement, emphasizing new or contrasting information, and conveying emotion [6]. Emotional prosody has been studied predominantly in people with right hemisphere disorders who may demonstrate emotional aprosodia, or the unique difficulty in either identifying or producing the tonal patterns that indicate emotional states. Emotional prosody has been localized to the amygdala, bilateral ventral striatum, anterior insula, temporal poles, and right posterior superior temporal perisulcal gyrus [7]. For some patients, deficits in emotional prosody production and comprehension co-occur, but this co-occurrence is not universal. For example, a 2003 case study described a woman with FTLD who appeared to have intact receptive emotional prosody in the presence of expressive affective aprosodia, affective prosoplegia, amusia, and deficits in automatic speech. Her atrophy localized to the right frontal cortical regions [8], and the authors suggested that the patient’s behavioral presentation was “analogous to primary progressive aphasia, but disrupting right frontal lobe-mediated functions.”

Deficits in emotional prosody can impair an individual’s social connectedness and ability to convey empathy significantly, thereby amplifying the difficulties associated with discourse for people with communication impairments. Progressive aprosodia is a condition that has been described in the literature independently of PPA [9]. Acoustic speech metrics have been examined across patients with nfavPPA and lvPPA. Word syllable duration is longer for patients with nfavPPA than lvPPA, though the authors emphasized the heterogeneity of their samples [10]. There is a modest literature specific to emotional aprosodia in patients with PPA. Matias-Guiu et al. [11] examined prosody in patients with PPA reading aloud and demonstrated that pitch variables, sentence duration, reading speed, and pauses could distinguish among PPA variants. Jiang et al. [12] conducted a study on emotional prosody recognition using normal and 6-, 12-, and 18-channel vocoded speech among individuals with AD and those with various PPA variants. Both patients with AD and PPA demonstrated impaired comprehension of emotional prosody in clear speech and when the signal was degraded, when compared to controls. Patients with lvPPA appeared to be most sensitive to signal quality and performed significantly worse than those with svPPA across vocoded trials. However, in most ways examined, people with PPA did not perform markedly worse than those with dementia without the PPA syndrome. Finally, Geraudie et al. [13] compared the fundamental frequency range of patients with frontotemporal neurodegeneration with and without svPPA and healthy controls. They found that expressive prosody correlated with grey matter volume in the right superior temporal lobe among patients with svPPA when controlling for age, sex, disease severity, and total intercranial volume.

In addition to conveying speaker sentiments, prior work has shown that speakers use prosodic information along with visual information [14] and other cues to support comprehension of words and sentences both when healthy speakers learn new languages [15,16,17,18] and when re-learning communication after stroke (for example, Melodic Intonation Therapy) [19,20,21,22,23,24,25]. However, there remain many questions regarding how acoustic and visual cues support communication among people with PPA. Compared to those with post-stroke aphasia, patients with PPA have unique difficulties detecting the correct emotions conveyed in photos [26,27,28]. Systematic manipulations of contextual support and the effects of specific cues on sentence-level comprehension have never been investigated in PPA, despite the importance of identifying effective means of supporting communication in this population.

The Hopkins Auditory Comprehension with Context Assessment (HACCA) was developed in 2022 [29,30] to examine the effect of paralinguistic and prosodic cues on sentence-level comprehension. In the HACCA, patients hear a sentence and must select the picture that best matches the sentence’s meaning. Sentences are presented with three levels of paralinguistic support: (1) no emotional prosody or observable facial cues of the speaker’s emotion, (2) congruent emotional prosody to the sentence meaning, without facial cues, and (3) congruent emotional prosody and facial cues consistent with the sentence meaning. Tilton-Bolowsky et al. [30] used this tool to show that sentence comprehension in people with post-stroke aphasia is facilitated by the presence of emotional prosodic cues, likely indicating reliance on the healthy right hemisphere to aid comprehension. However, given that people with PPA also have neurodegeneration in the right hemisphere, they might be unable to rely on prosodic cues. Instead, they might still rely on visual cues, which are predominantly processed in bilateral occipitoparietal regions, areas not typically affected in PPA.

The aim of the present study was to test the hypothesis that some people with PPA may have comprehension deficits that are not facilitated by prosodic cues. We also expected that patients with the three most common PPA variants would differ in the extent to which hearing and seeing congruent emotional prosody and facial expressions supported their sentence comprehension on the HACCA. As the HACCA is a relatively new instrument, an exploratory aim was to examine the relationship between HACCA performance under the different cueing conditions and established measures of communication, cognition, and behavior.

## 2. Materials and Methods

### 2.1. Records Reviewed

English-speaking patients with PPA received the HACCA as part of standard-of-care outpatient cognitive and neurological evaluations conducted by author AEH, as well as through research protocols recruiting individuals with established PPA at Johns Hopkins University School of Medicine between February 2021 and September 2025. All work was conducted under approval by the Johns Hopkins University Institutional Review Board. The approval specified that informed consent was not required to collect these data, as all measures were either part of the standard of care or part of a PPA treatment study, which included a separate consent form. Records included for analysis were those of adult patients who received (either in that visit or during a prior visit) a diagnosis of one of the three main variants of PPA based on a review of history, communication and cognitive performance, and the results of neuroimaging, consistent with the consensus criteria outlined in Gorno-Tempini et al. [1]. Classification relied on a consistent pattern of atrophy on MRI affecting left superior temporal-inferior parietal gyri in lvPPA, left inferior frontal and insula in nfavPPA, and left inferior temporal gyrus and temporal pole in svPPA. Records were excluded if the individual had uncorrected visual or hearing acuity impairment by self/caregiver report or medical records, neurologic disease affecting the brain other than PPA, or previous developmental or neuropsychiatric disorders affecting communication.

A total of 88 participants who met the criteria for PPA completed the HACCA during their evaluation. Of these, one participant had an unclassifiable subtype and was removed from statistical analyses. Sample characteristics are summarized in Table 1. Two logopenic patients did not receive the video condition.

Additional cognitive testing was available for 82 participants, though not all patients completed all measures, as provided below. Individual components are selected by the neurologist. Patients are encouraged to take necessary breaks to complete all testing requested. However, patients or caregivers may terminate testing at any time for any reason. Testing includes select subtests from the National Alzheimer’s Coordinating Center (NACC) Uniform Dataset (UDS) standardized evaluation battery frontotemporal lobar degeneration (FTLD) module version 2.0 (https://files.alz.washington.edu/documentation/ftld2-ivp-packet.pdf, accessed on 28 January 2026): Benson Figure Copy (17 points each), verbal fluency, Regular & Irregular Word Tests—Reading & Spelling to Dictation (15 points each), Sentence Repetition Test (5 items), Sentence Reading Test (5 items), and Noun & Verb Naming Subtests—Oral and Written Modalities (16 points each). Additional measures are included in the cognitive testing battery to further evaluate language generation. These include the Boston Naming Test [31], Hopkins Action Naming Assessment [32] (1 point per item, 30 points total), Pyramids and Palm Trees test [33,34] (1 point per item, 14 points total), Kissing and Dancing test [35] (1 point per item, 14 points total), expanded sentence reading and repetition [36], the Morphosyntactic Generation test [37], Berndt Picture-Word Verification of Nouns and Verbs [38] (1 point per item, 30 points total), and the modified Frontal Behavioral Inventory (mFBI; [39,40]). A full description of this battery has been reported previously in the literature [41].

### 2.2. Assessing Prosody in Patients with PPA

**Figure 1:**
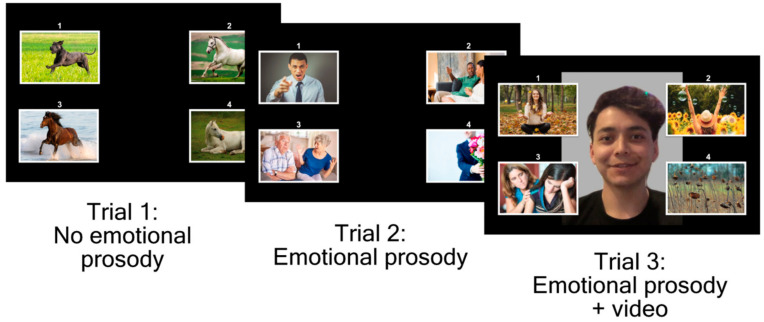
HACCA items. Patients select the picture that aligns with the sentence that they hear read aloud.

During the HACCA, patients hear a sentence or short story (up to 3 sentences) and are asked to select the best picture to match the meaning of a sentence from a field of four (Figure 1). There are three types of trials: (1) the sentences are produced with no emotional prosody or observable facial cues of the speaker’s emotion, (2) the sentences are produced with congruent emotional prosody to the sentence meaning, and (3) the sentences are produced with congruent emotional prosody along with a video of the speaker including facial cues consistent with the emotional content of the sentence. All sentences and videos feature the same speaker. The sentences vary in length and complexity, and patients do not receive feedback on accuracy beyond a brief two-item training period. As the task is intentionally designed to identify functional deficits, accuracy among healthy speakers generally approaches ceiling.

In the previously established version of the HACCA, the three types of trials are presented in sequential blocks of increasing support using the same 10 sentences, which are presented in order of length and grammatical complexity. First, responders hear the sentences without emotional cues, then with emotionally appropriate cues, and finally with both emotional cues and visual cues. If a given participant completes the first block (no cues) with 100% accuracy, they do not receive additional blocks, as it is assumed they would do so with similarly maximal accuracy (i.e., it was assumed that the lowest cue level was the most difficult).

When the assessment was deployed in individuals with PPA, there were concerns that performance could be influenced by practice effects or strategic behaviors when sentences were presented a second or third time. Moreover, it was less certain from the available evidence that greater cues would result in consistently higher accuracy across PPA variants. In response, a modified administration of the HACCA was employed in 15 patients. In the modified administration, the levels of support were presented in a random order instead of in blocks, and the stopping rule was removed. When performance by variant on the established version was compared to performance on the modified version, accuracy was statistically similar overall and when each subtype was considered independently (Table 2). Thus, data from the two versions were combined for all subsequent analyses (data separated by version are available in Appendix A).

### 2.3. Statistical Analysis

Prior work using the HACCA demonstrated that many patients with limited language deficits score at or near ceiling [30]. Moreover, it has been established that individuals with semantic PPA generally perform the most poorly on language assessments such as the HACCA that require precise word knowledge, while individuals with non-fluent PPA perform the best, and those with logopenic PPA tend to perform in the middle [41,42]. Thus, the investigation was not designed to statistically examine comparisons across variants in overall accuracy, but rather to examine individual patterns of performance across cueing conditions, and whether these patterns varied as a function of variant.

HACCA performance was classified using the following taxonomy applied in order:

1. Ceiling: 9+ accuracy out of 10 on all three levels of support;
2. Benefitting from prosody (Prosody+): at least a 2-point improvement between no cue and prosody only conditions;
3. Benefitting only when video was introduced (Video+): at least a 2-point difference between prosody only and prosody plus video conditions;
4. Worsening with additional support (Cue−): at least 2 points fewer when either prosody or video was added compared to no cues;
5. No change due to cues (∅): incremental increases or decreases in cues resulted in no more than 1 point in change in accuracy.

The threshold for operationalizing change using 2 points was based upon the observation that among a previously reported sample of controls, no participant varied more than one point across conditions [30]. The *a priori* statistical plan was a chi-square test. However, 8/15, or 53%, of the expected cell frequencies (Table 3) were below 5, which threatens one of the assumptions of the chi-square statistic. A common guideline is that no more than 20% of cells should be expected to be below 5 [43]. Therefore, a Fisher’s exact test (Fisher–Freeman–Halton) was used. Statistical analyses were carried out in R using the Fisher.test function and a significance of α = 0.05. Cramér’s V was calculated using Stata 19. Those within each performance group were examined individually to investigate patterns of performance classification by variant. Exploratory Spearman’s ρ correlational analyses were conducted in R using cor and corrplot and were not subject to hypothesis testing.

## 3. Results

### 3.1. Sample Characteristics

**Figure 2:**
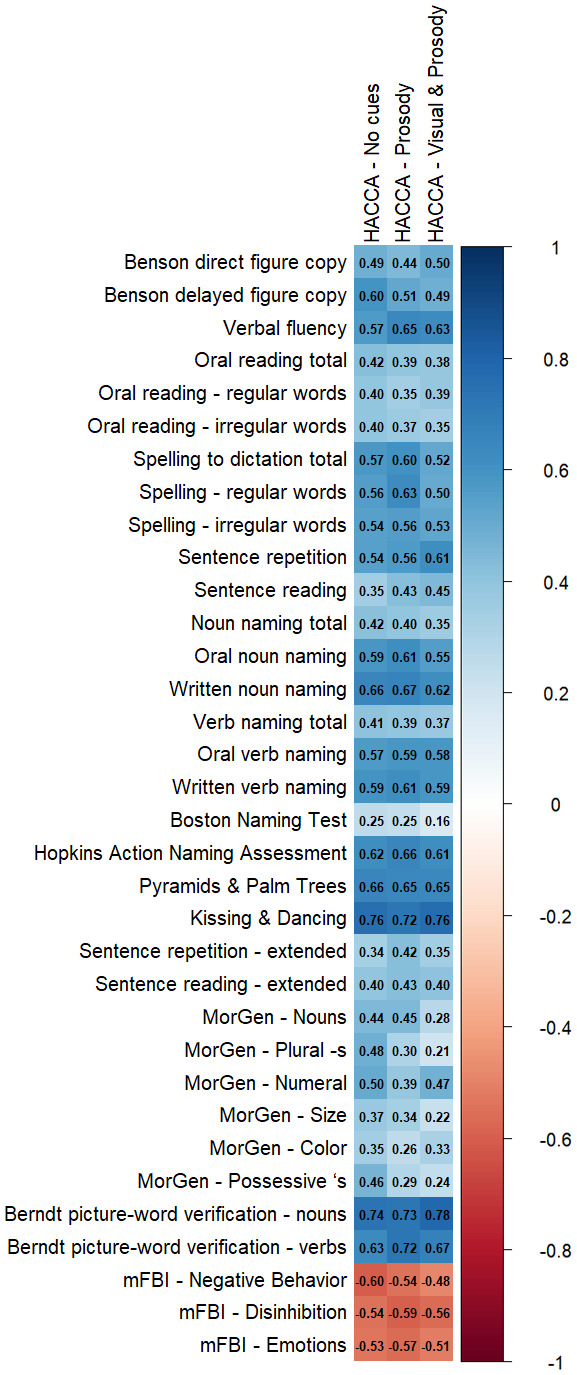
Correlations between HACCA performance and other assessments in the cognitive-communication and behavioral battery.

Cognitive battery performance by the PPA variant is detailed in Table 4. All but five participants received the HACCA on the same day as the remaining cognitive testing, and those remaining received it within six months. Pursuant to the exploratory aim to understand HACCA performance in the broader context of the individual’s communication, cognition, and behavior, correlation values are presented in Figure 2.

### 3.2. HACCA Performance by PPA Variant (Group Statistics)

**Figure 3:**
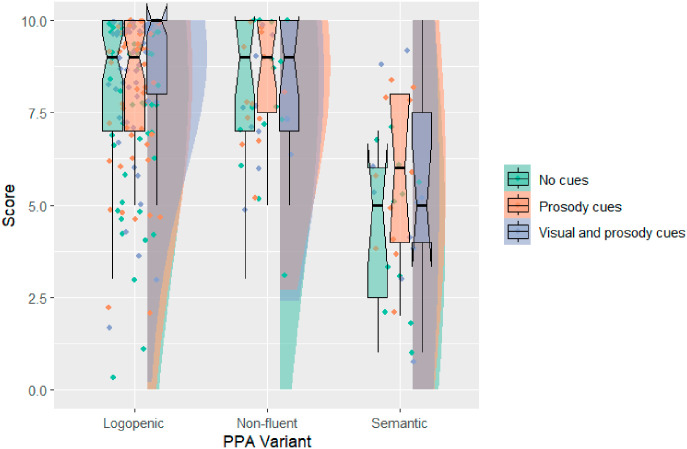
HACCA performance by PPA variant. Notches represent 95% confidence intervals.

Overall task performance is summarized in Figure 3. As anticipated, the data demonstrate strong negative skewness associated with the anticipated ceiling effects.

**Figure 4:**
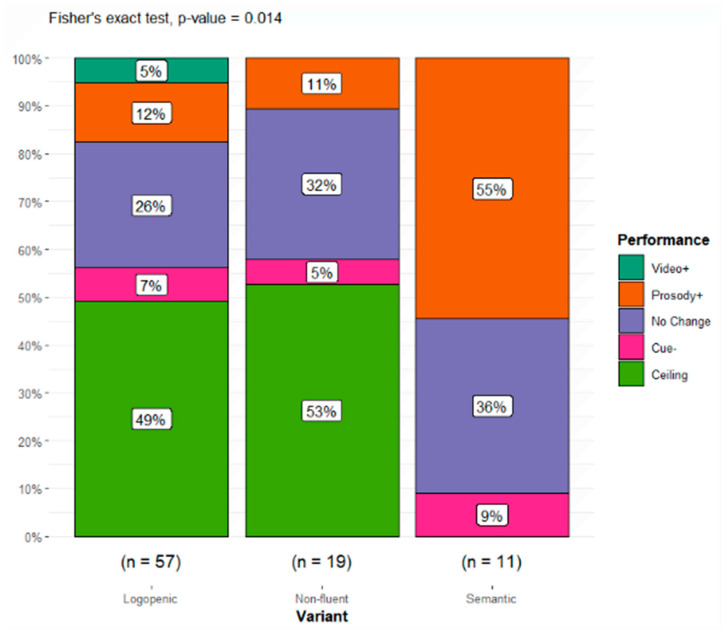
Performance classifications as a proportion of the sample by PPA variant.

Individual performance categories were distributed among PPA variants (Table 5 and Figure 4). The overall significance for the 5 × 3 table was *p* = 0.014, demonstrating that the distribution of performance classifications was influenced significantly by the PPA variant. The effect size was large when accounting for degrees of freedom [44], φ*_c_* = 0.32. Patients who benefitted from prosody often did so, resulting in ceiling performance that persisted regardless of the addition of video. Many of those with no clear response to cues also demonstrated high accuracy across conditions (see Appendix B for breakdown of ceiling performance by condition and variant, and for standardized residuals).

As anticipated, the majority of patients with lvPPA and nfavPPA either performed the HACCA at ceiling regardless of the cueing condition or did not demonstrate changes in performance due to increased cues. When patients with svPPA benefited from cues, this was most common in the addition of prosodic cues, with 55% showing enhanced discourse comprehension. Only 5% of lvPPA patients and 7% of nfavPPA patients performed worse with added cues, while no change was observed for the remaining 26% lvPPA and 32% nfavPPA patients.

### 3.3. Individual Profiles of HACCA Performance

Patients were examined individually to better understand the profiles associated with each performance classification (Table 6). Among the patients who benefited from prosody over no cues, there was a wide range of accuracies and demographics. This performance classification also included the individual with an unclassifiable variant of PPA. The three patients with logopenic PPA who benefitted only when video was introduced were not discernibly similar in other ways. Two of the three had poor performance in the no cue and prosodic cue conditions, while the third was one point shy of classification as ceiling performance overall.

Three participants who improved by at least two points over their performance with no cues when prosody was included also changed when video was included. Two performed worse when video was included (meeting the −2 criteria for Cue−; Table 6 ^†^), and one improved (meeting criteria for Video+; Table 6 ^‡^). Others remained within one point of the score with prosodic cues when video was included. Among those whose accuracy was lower when either cue was added (Cue−), three patients with logopenic variant PPA (P21–23) worsened with prosody compared to no cues. Two patients, one with the non-fluent variant and one with the semantic variant, worsened with video only.

## 4. Discussion

The aim of this investigation was to examine how prosodic and visual cues support sentence comprehension in patients with PPA. Given the modest, mottled landscape of prior work regarding emotional prosody and emotional processing in PPA and the relatively recent development of the HACCA instrument, our hypothesis was that, while PPA variants would differ in performance, individuals would also differ in their utilization of these cues in ways that may not be best captured by group performance by variant. We also sought to provide a preliminary evaluation of the concurrent validity of the HACCA within this population, using established tasks within and beyond the NACC UDS FLTD battery as gold standards for cognitive-communication evaluation.

As anticipated based on the prior work in stroke, most patients with logopenic and nonfluent PPA performed at or near ceiling on the HACCA, while individuals with semantic PPA demonstrated greater difficulty and variability (though median performance remained around 50%, well above chance). There was a statistically significant difference in the distribution of responses to cue conditions as a function of variant. Most patients who were not at ceiling without cues experienced a positive effect from the addition of prosody. Yet, individuals within each cue-dependent performance classification varied considerably in both overall accuracy and demographic factors.

Our findings for svPPA are notable, although this variant was the smallest group. More than half of the participants who showed a differential performance by cue condition demonstrated improvement in sentence-level comprehension in the presence of prosodic cues. Interestingly, in the Jiang et al. study, which focused on the comprehension of noise-vocoded conditions on individuals with PPA and AD, individuals with lvPPA performed significantly worse than those with svPPA on comprehension of acoustically degraded speech [12]. Similarly, in our study, a few people with lvPPA performed worse with prosodic/facial cues than with no cues. It is possible that they focused only on the cues, rather than the sentences, and selected items consistent with the emotion (but not other content). Two participants improved with prosody but were less accurate with video, and the two were less accurate with video, even when prosody did not affect accuracy. None of the participants with lvPPA showed either pattern, potentially indicating that they were less distracted by the cues.

We also observed that HACCA performance had a strong monotonic relationship with multiple assessments of language, including the verification of single nouns and verbs. As with the HACCA, picture–word verification does not require patients to generate language. In the Berndt picture–word verification test, patients are presented with a picture and asked to respond “yes” or “no” to whether it matches a given label (e.g., the patient is shown a picture of a “doctor” and asked, “Is this a daughter?”). HACCA items are presented within a four-item forced choice of images representing the meaning of a sentence, but imageable aspects of sentences are limited to environments, subjects, and actions—that is, frequently, nouns and verbs. High concurrent validity was noted between the HACCA and two other forced-choice measures of language understanding, the Pyramids and Palm Trees and Kissing and Dancing tests, which probe semantic associations between sets of nouns and verbs, respectively. These findings highlight the influence of response modality in performance, both generally and among patients with PPA specifically.

## 5. Limitations

As PPA is an uncommon syndrome, a key limitation of our study was both modest and unbalanced samples from each variant. This limited the statistical approaches that could be rigorously pursued and was a barrier to examining the co-occurrence of right hemisphere neurodegeneration among the patients in our sample, which may have provided a clear mechanistic account of the differences in the benefits derived from emotional prosody. Acquiring and analyzing neuroimages within our sample is a crucial future direction. There also remain unanswered psychometric questions about the use of the HACCA as an instrument, especially across linguistic communities. The HACCA was designed with low testing burden as a paramount consideration due to the fact that many patients with PPA and dementia become fatigued with extensive cognitive testing. The brevity of the assessment and sample size also contributed to limitations in the ability to examine social and dialectal factors that influence emotional prosody [45]. Cultural and sociological factors influence how emotions are conveyed, both visually in body language and facial expression, and acoustically [46]. It is possible that the number of items in the HACCA may also constitute insufficient evidence to appropriately capture difficulties or change. It is worth noting that more than half of the testing domains probed in the National Alzheimer’s Coordinating Center Uniform Data Set FTLD Battery use fewer than 10 items, and these assessments remain effective in distinguishing among PPA variants both when used separately [41] and together [47]. The sentence-level tests within the Battery, which probe repetition, reading, and grammar, all use fewer than 10 items. Nevertheless, we acknowledge the lack of rigorous psychometric evaluation in this test (and in the UDS FTLD Battery). Finally, it is possible that the incidence of poorer performance given additional cues is under-reported in the present design due to the assumption that ceiling performance in block 1 would not change with the addition of cues (for the subset for whom we made this assumption early in this study). Given these limitations, the finding should be considered preliminary and serve as a springboard for further study as described below.

## 6. Conclusions

Despite these limitations, there is some preliminary evidence to suggest that congruent prosodic and visual information may support sentence comprehension for individuals with svPPA. Important future directions include recruiting a larger and more proportional sample that has available neuroimaging data, such that complementary questions may be answered regarding the use of visual and prosodic emotional cues and specific regions of atrophy. Item-level error analyses may provide valuable new insights into not only what supports accurate comprehension for patients with different PPA variants, but also what information specifically proves to be an effective foil, as this was something that could only be speculated about in the present retrospective review. For example, it may be that images with similar subjects to the target are consistently effective foils regardless of whether other aspects of the pictured event are congruent (e.g., for the sentence “A cross marked her daughter’s grave,” a picture of a young girl may be an effective foil even if she is shown dancing). In the other extreme, patients may be more likely to pair sentences with images with the appropriate emotional valence, even if the subject and pictured actions are not specific to the sentence content or depict conflicting information (e.g., selecting a foil of a child crying over her dog, which, while sad, does not pertain to the content of the sentence). This evaluation of error patterns is likely to provide therapeutically useful insights into strategies patients use when adaptively responding in real-world conversational contexts.

## Figures and Tables

**Table 1:** Sample characteristics.

	lvPPA	nfavPPA	svPPA
N	57	19	11
Age (years)	71 ± 8	71 ± 6	69 ± 6
Sex F:M	30:27	12:7	8:3
Race			
White	53	17	9
Black	3	1	1
Other	1	1	1
Ethnicity			
Hispanic	2	0	0
Non-Hispanic	54	19	11
Other	1	0	0

Values are reported as mean ± standard deviation or count.

**Table 2:** Significance values (*p*) from simple *t*-tests across HACCA versions.

	No Cues	Prosody	Visual & Prosody
Logopenic	0.10	0.74	0.79
Non-fluent	0.27	0.33	0.26
Semantic	0.52	0.81	0.59
Overall	0.34	0.95	0.99

**Table 3:** Expected classification values.

	Ceiling	Prosody+	Video+	Cue−	∅
Logopenic	24.9	9.8	2	3.9	16.4
Non-fluent	8.3	3.3	0.7	1.3	5.5
Semantic	4.8	1.9	0.4	0.8	3.2

**Table 4:** Results of cognitive and behavioral evaluation.

	lvPPA		nfavPPA		svPPA		Total	
	N	Score	N	Score	N	Score	N	Score
HACCA—No cues (/10)	57	8 ± 2	19	8 ± 2	11	4 ± 2	87	8 ± 3
HACCA—Prosody (/10)	57	8 ± 2	19	9 ± 2	11	6 ± 2	87	8 ± 2
HACCA—Visual & Prosody (/10)	55	9 ± 2	19	8 ± 2	11	6 ± 3	85	8 ± 2
**FTLD Module subtests**								
Benson direct figure copy (/17)	45	14 ± 4	12	14 ± 4	9	12 ± 5	66	14 ± 4
Benson delayed figure copy (/17)	43	8 ± 4	12	10 ± 5	9	3 ± 5	64	7 ± 5
Verbal fluency	43	12 ± 9	11	8 ± 4	8	8 ± 7	62	11 ± 8
Oral reading total (/30)	47	27 ± 5	11	26 ± 3	9	25 ± 5	67	26 ± 5
Regular words (/15)	47	14 ± 2	11	15 ± 1	9	14 ± 1	67	14 ± 2
Irregular words (/15)	47	12 ± 3	11	12 ± 2	9	11 ± 4	67	12 ± 3
Spelling to dictation total (/30)	40	21 ± 9	9	23 ± 8	7	16 ± 10	56	21 ± 9
Regular words (/15)	40	12 ± 5	9	13 ± 3	7	10 ± 6	56	12 ± 5
Irregular words (/15)	40	9 ± 5	9	10 ± 5	7	6 ± 5	56	9 ± 5
Sentence repetition (/5)	56	3 ± 2	12	2 ± 2	9	2 ± 1	77	2 ± 2
Sentence reading (/5)	55	4 ± 1	13	3 ± 2	9	4 ± 2	77	4 ± 2
Noun naming total (/32)	55	21 ± 13	16	25 ± 10	10	16 ± 10	81	21 ± 12
Oral noun naming (/16)	44	13 ± 4	14	15 ± 1	9	9 ± 4	67	13 ± 4
Written noun naming (/16)	42	13 ± 5	14	14 ± 3	9	8 ± 5	65	12 ± 5
Verb naming total (/32)	55	19 ± 12	16	21 ± 11	10	14 ± 9	81	18 ± 12
Oral verb naming (/16)	43	13 ± 4	14	13 ± 3	9	9 ± 4	66	12 ± 4
Written verb naming (/16)	41	12 ± 4	14	11 ± 4	9	6 ± 5	64	11 ± 5
**Additional Instruments**								
Boston Naming Test (/60)	38	37 ± 15	7	45 ± 10	2	16 ± 11	47	37 ± 15
Hopkins Action Naming Assessment (/30)	40	17 ± 7	8	21 ± 5	6	6 ± 2	54	16 ± 8
Pyramids & Palm Trees (/14)	42	13 ± 2	11	13 ± 1	9	10 ± 3	62	13 ± 2
Kissing & Dancing (/15)	35	11 ± 3	8	12 ± 3	7	8 ± 4	50	11 ± 3
Sentence Repetition—extended (/5)	52	0 ± 1	12	1 ± 1	9	0 ± 0	73	0 ± 1
Sentence Reading—extended (/5)	53	3 ± 2	13	2 ± 2	9	3 ± 2	75	3 ± 2
Morphosyntactic Generation								
Nouns (/60)	18	56 ± 14	8	60 ± 1	2	27 ± 38	28	55 ± 16
Plural-s (/31)	18	27 ± 8	8	31 ± 1	2	12 ± 16	28	27 ± 8
Numeral (/8)	18	7 ± 2	8	7 ± 1	2	4 ± 5	28	6 ± 2
Size (/16)	18	9 ± 5	8	15 ± 1	2	1 ± 1	28	10 ± 5
Color (/19)	18	13 ± 7	8	18 ± 3	2	0 ± 0	28	14 ± 7
Possessive ’s (/17)	18	12 ± 7	8	15 ± 6	2	0 ± 0	28	12 ± 7
Berndt picture–word verification—nouns (/30)	39	27 ± 4	6	29 ± 1	8	19 ± 10	53	26 ± 6
Berndt picture–word verification—verbs (/30)	30	27 ± 3	6	29 ± 1	5	21 ± 6	41	26 ± 4
mFBI—Negative Behavior (/27)	13	11 ± 7	3	10 ± 10	5	13 ± 7	21	11 ± 7
mFBI—Disinhibition (/39)	13	5 ± 5	3	2 ± 3	5	8 ± 7	21	5 ± 6
mFBI—Emotions (/12)	13	2 ± 3	3	2 ± 3	5	2 ± 2	21	2 ± 2

**Table 5:** Classification by performance pattern.

	Ceiling	Prosody+ ^1^	Video+ ^2^	Cue− ^3^	∅ ^4^	Total
Logopenic	28	7	3	4	15	57
Non-fluent	10	2	0	1	6	19
Semantic	0	6	0	1	4	11
Total	38	15	3	6	25	87

^1^ Prosody+: Benefitting from prosody. ^2^ Video+: Benefitting only when video was introduced. ^3^ Cue−: Worsening with additional cues. ^4^ ∅: No change.

**Table 6:** Individual profiles of HACCA performance.

Patient Characteristics				Performance by Cue			
Code	Sex	Age	PPA Type	None	Prosody	Video	Category
P1	M	55–59	Logopenic	6	8	8	Prosody+
P2	F	55–59	Logopenic	0	2	3	Prosody+
P3	M	65–69	Logopenic	8	10	10	Prosody+
P4	M	70–74	Logopenic	7	10	10	Prosody+
P5	F	75–79	Logopenic	5	9	8	Prosody+
P6	M	80–84	Logopenic	4	7	7	Prosody+
P7	F	85+	Logopenic	3	5	4	Prosody+
P8 ^†^	M	70–74	Non-fluent	5	7	5	Prosody+
P9	M	75–79	Non-fluent	3	7	7	Prosody+
P10 ^†^	F	60–64	Semantic	1	4	1	Prosody+
P11	F	65–69	Semantic	3	5	5	Prosody+
P12	F	70–74	Semantic	3	6	5	Prosody+
P13	M	70–74	Semantic	6	8	9	Prosody+
P14	F	75–79	Semantic	2	4	4	Prosody+
P15 ^‡^	F	75–79	Semantic	6	8	10	Prosody+
P16	M	60–64	Unclassified	4	9	9	Prosody+
P17	M	60–64	Logopenic	9	8	10	Video+
P18	M	75–79	Logopenic	5	5	8	Video+
P19	M	80–84	Logopenic	4	5	8	Video+
P20	M	70–74	Logopenic	9	7	9	Cue−
P21	F	75–79	Logopenic	7	5	5	Cue−
P22	F	75–79	Logopenic	8	6	7	Cue−
P23	F	75–79	Logopenic	10	8	9	Cue−
P24	F	70–74	Non-fluent	8	9	6	Cue−
P25	F	60–64	Semantic	7	8	6	Cue−

^†^ Participants who improved by at least two points over their performance with no cues when prosody was included, but performed worse when video was included. ^‡^ Participants who improved by at least two points over their performance with no cues when prosody was included, and improved again when video was included.

## Data Availability

The data presented in this study are available on request from the corresponding author due to restrictions imposed by the Data Trust committee of Johns Hopkins University School of Medicine.

## Abbreviations

The following abbreviations are used in this manuscript:

AD	Alzheimer’s disease
FTLD	Frontotemporal lobar degeneration
HACCA	Hopkins Auditory Comprehension with Context Assessment
PPA	Primary progressive aphasia

## Appendix A

**Table A1:** Performance classification separated by HACCA version.

	Ceiling	Prosody+ ^1^	Video+ ^2^	Cue− ^3^	∅ ^4^	Total
HACCA Version 1: Sequential administration						
Logopenic	23	7	3	2	14	49
Non-fluent	8	1	0	1	5	15
Semantic	0	5	0	0	3	8
Total	**31**	**13**	**3**	**3**	**22**	**72**
HACCA Version 2: Randomized administration						
Logopenic	5	0	0	2	1	8
Non-fluent	2	1	0	0	1	4
Semantic	0	1	0	1	1	3
Total	**7**	**2**	0	**3**	**3**	**15**
**Grand** **Total**	**38**	**15**	**3**	**6**	**25**	**87**

^1^ Prosody+: Benefitting from prosody. ^2^ Video+: Benefitting only when video was introduced. ^3^ Cue−: Worsening with additional cues. ^4^ ∅: No change. As the majority of participants received the original version, results of HACCA Version 1 in isolation continue to reach significance, *p* = 0.04, while those for HACCA Version 2 do not.

## Appendix B

**Table A2:** Ceiling performance by PPA variant and task condition.

	No Cues	Prosody Cues	Visual & Prosody Cues
Logopenic	31/57 (54%)	33/57 (58%)	37/57 (65%)
Non-fluent	10/19 (53%)	11/19 (58%)	10/19 (53%)
Semantic	0/11 (0%)	0/11 (0%)	3/11 (27%)

**Table A3:** Standardized residuals for Fisher-Freeman-Halton test.

	Ceiling	Prosody+ ^1^	Video+ ^2^	Cue− ^3^	∅ ^4^
Logopenic	0.62	−0.89	0.71	0.05	−0.35
Non-fluent	0.59	−0.72	−0.84	−0.26	0.21
Semantic	−2.19	2.97	−0.63	0.22	0.45

^1^ Prosody+: Benefitting from prosody. ^2^ Video+: Benefitting only when video was introduced. ^3^ Cue−: Worsening with additional cues. ^4^ ∅: No change.
